# Graphormer supervised *de novo* protein design method and function validation

**DOI:** 10.1093/bib/bbae135

**Published:** 2024-03-31

**Authors:** Junxi Mu, Zhengxin Li, Bo Zhang, Qi Zhang, Jamshed Iqbal, Abdul Wadood, Ting Wei, Yan Feng, Hai-Feng Chen

**Affiliations:** State Key Laboratory of Microbial metabolism, Joint International Research Laboratory of Metabolic Developmental Sciences, Department of Bioinformatics and Biostatistics, National Experimental Teaching Center for Life Sciences and Biotechnology, School of Life Sciences and Biotechnology, Shanghai Jiao Tong University, 800 Dongchuan Road, Shanghai, 200240, China; Center for Life Sciences, Academy for Advanced Interdisciplinary Studies, Peking University, No.5 Yiheyuan Road, Beijing, 100871, China; State Key Laboratory of Microbial metabolism, Joint International Research Laboratory of Metabolic Developmental Sciences, Department of Bioinformatics and Biostatistics, National Experimental Teaching Center for Life Sciences and Biotechnology, School of Life Sciences and Biotechnology, Shanghai Jiao Tong University, 800 Dongchuan Road, Shanghai, 200240, China; State Key Laboratory of Microbial metabolism, Joint International Research Laboratory of Metabolic Developmental Sciences, Department of Bioinformatics and Biostatistics, National Experimental Teaching Center for Life Sciences and Biotechnology, School of Life Sciences and Biotechnology, Shanghai Jiao Tong University, 800 Dongchuan Road, Shanghai, 200240, China; State Key Laboratory of Microbial metabolism, Joint International Research Laboratory of Metabolic Developmental Sciences, Department of Bioinformatics and Biostatistics, National Experimental Teaching Center for Life Sciences and Biotechnology, School of Life Sciences and Biotechnology, Shanghai Jiao Tong University, 800 Dongchuan Road, Shanghai, 200240, China; Centre for Advanced Drug Research, COMSATS University Islamabad, Abbottabad Campus, Abbottabad, 22060, Pakistan; Department of Biochemistry, Abdul Wali Khan University Mardan, Mardan, 23200, Pakistan; State Key Laboratory of Microbial metabolism, Joint International Research Laboratory of Metabolic Developmental Sciences, Department of Bioinformatics and Biostatistics, National Experimental Teaching Center for Life Sciences and Biotechnology, School of Life Sciences and Biotechnology, Shanghai Jiao Tong University, 800 Dongchuan Road, Shanghai, 200240, China; State Key Laboratory of Microbial metabolism, Joint International Research Laboratory of Metabolic Developmental Sciences, Department of Bioinformatics and Biostatistics, National Experimental Teaching Center for Life Sciences and Biotechnology, School of Life Sciences and Biotechnology, Shanghai Jiao Tong University, 800 Dongchuan Road, Shanghai, 200240, China; State Key Laboratory of Microbial metabolism, Joint International Research Laboratory of Metabolic Developmental Sciences, Department of Bioinformatics and Biostatistics, National Experimental Teaching Center for Life Sciences and Biotechnology, School of Life Sciences and Biotechnology, Shanghai Jiao Tong University, 800 Dongchuan Road, Shanghai, 200240, China

**Keywords:** protein sequence design, Graphormer architecture, GPD model, function validation

## Abstract

Protein design is central to nearly all protein engineering problems, as it can enable the creation of proteins with new biological functions, such as improving the catalytic efficiency of enzymes. One key facet of protein design, fixed-backbone protein sequence design, seeks to design new sequences that will conform to a prescribed protein backbone structure. Nonetheless, existing sequence design methods present limitations, such as low sequence diversity and shortcomings in experimental validation of the designed functional proteins. These inadequacies obstruct the goal of functional protein design. To improve these limitations, we initially developed the Graphormer-based Protein Design (GPD) model. This model utilizes the Transformer on a graph-based representation of three-dimensional protein structures and incorporates Gaussian noise and a sequence random masks to node features, thereby enhancing sequence recovery and diversity. The performance of the GPD model was significantly better than that of the state-of-the-art ProteinMPNN model on multiple independent tests, especially for sequence diversity. We employed GPD to design CalB hydrolase and generated nine artificially designed CalB proteins. The results show a 1.7-fold increase in catalytic activity compared to that of the wild-type CalB and strong substrate selectivity on *p*-nitrophenyl acetate with different carbon chain lengths (C2–C16). Thus, the GPD method could be used for the *de novo* design of industrial enzymes and protein drugs. The code was released at https://github.com/decodermu/GPD.

## INTRODUCTION

Protein design is a fundamental aspect of protein engineering with extensive applications, such as enzyme engineering, which aims to create designed enzymes with enhanced catalytic efficiency [1], and therapeutic applications that focus on designing immune proteins with increased therapeutic affinity [2]. One key method in this field is *de novo* protein design, which involves creating novel amino acid sequences that encode proteins with the desired properties [3]. *De novo* protein design can be divided into two primary tasks: protein backbone design and sequence design. This paper focuses on the fixed-backbone protein sequence design, also known as the inverse protein folding problem. The goal is to generate novel sequences that fold into the fixed-backbone structure. However, the designed sequences must not only be structurally compatible with the intended backbone but also functionally active, exhibiting the specified properties [4].

Numerous studies have been conducted on protein sequence design, and the primary approaches for fixed-backbone protein sequence design generally fall into two categories: classical physical principle–based protein sequence design and deep learning–based protein sequence design [5, 6]. Classical physical principle–based protein design, exemplified by the popular protein design framework Rosetta [7], aims to minimize the parametric energy function of the target structure. This is achieved by searching for the optimal combination of sequence and conformations [3]. However, the effectiveness of these classical physical principle–based approaches relies heavily on the accuracy of the energy functions for protein physics and the efficiency of the sampling algorithms. This suggests that there are opportunities for improving both the accuracy and computational speed of these methods [8]. The swift advancement of deep learning technology has facilitated the emergence of deep learning–based protein design. Deep learning–based protein sequence design not only accelerates the design process with high accuracy but also revolutionizes the field by capturing complex patterns in protein data [8]. Supplementary Figure S1 presents an overview of all the deep learning–based protein sequence design methods to date [9–21].

However, experimental examinations of the designed protein sequences have only been reported by a few methods so far. The sequences designed by 3DCNN [15], ABACUS-R [16] and ProteinMPNN [18] have been examined experimentally using crystallography. Furthermore, sequences from ProteinSolver [13] and ProDESIGN-LE [21] have demonstrated the desired secondary structure contents, evidenced by circular dichroism signatures. Despite these promising results, the aforementioned methods face limitations in protein functional validation and sequence diversity, failing to meet the demands of functional protein design. While a handful of methods have reported experimental structures of designed sequences [13, 15, 16, 18, 21], none has been used to analyze the functionality of these sequences. Ideally, designed sequences should surpass the performance of their wild-type proteins.

Furthermore, existing methods have primarily focused on improving sequence recovery to their native counterparts. This focus has often led to a compromise in the exploration of sequence diversity, yielding overly uniform sequences that lack necessary variation. Enhancing the diversity of designed sequences is biologically significant for two reasons. Firstly, uniformity among designed sequences means that if one sequence fails in experimental validation, it could be indicative of a broader issue affecting similar sequences. Therefore, selecting more diverse sequences could improve the success rate of functional experiments. Secondly, a greater variety of designed sequences allows for a broader exploration of the sequence space landscape, which is crucial for advancing our understanding of protein functions.

In this study, we introduced the Graphormer-based Protein Design (GPD) toolbox, an innovative approach inspired by Graphormer [22]. This tool applies the Transformer model to a graph-based representation of three-dimensional (3D) protein structures for protein sequence design, incorporating a normally distributed random matrix into node features to augment sequence diversity. To improve the success rate in experimental outcomes, we implemented functional filtering based on criteria such as structure folding, solubility and function. Utilizing the GPD toolbox, we designed 1 million *de novo* sequences of CalB hydrolase. After functional filtering, nine sequences were selected for wet lab experiments. The experimental results show that the solubility of the nine designed sequences was 55.6%. Additionally, one of these designed sequences exhibited a remarkable 1.7-fold improvement in the catalytic activity compared with the CalB wild type. The GPD toolbox was publicly available at https://yu.life.sjtu.edu.cn/ChenLab/GPDGenerator/. This web server provided users with an automated platform for generating protein sequences based on given 3D protein structures.

## RESULTS

### The GPD architecture

**Figure 1:**
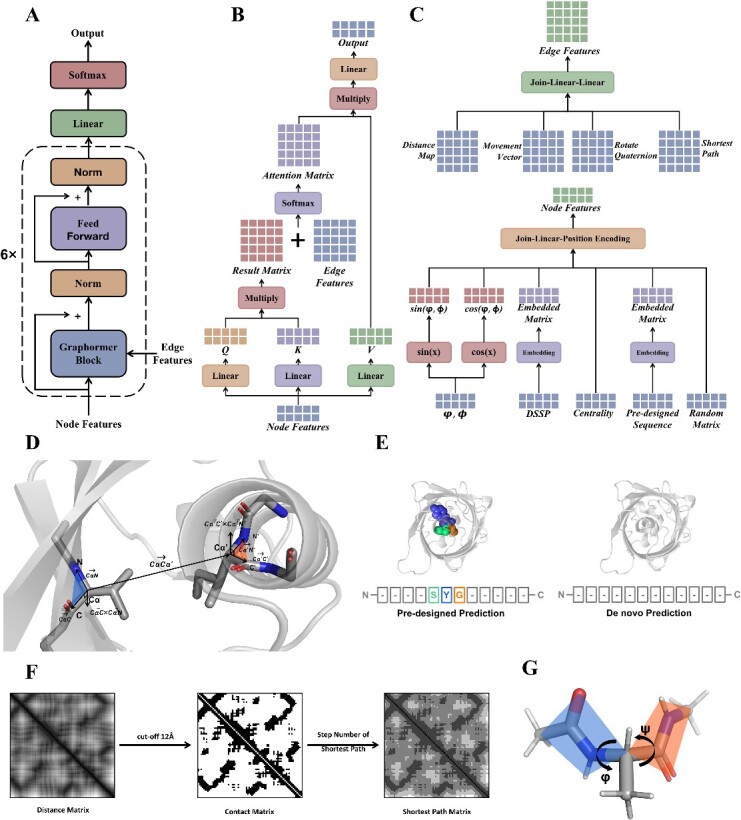
The GPD architecture. (**A**) The overall architecture of GPD. (**B**) The architecture of the Graphormer block. (**C**) The embedding process of edge features and node features. (**D**) The calculation process of distance map, local movement vector and rotate quaternion. (**E**) Two different ways of sequence prediction. (**F**) The calculation of the shortest pathway matrix. (**G**) The dihedral angles of residual backbone.

The GPD model directly employs the Transformer model to a graph-based representation of 3D protein structures (Figure 1). To enhance the diversity of the designed protein sequences, the GPD model incorporates a normally distributed random matrix into the node features. These node features comprise the main-chain dihedral angle, the secondary classification, the centrality of each residue, the pre-designed protein sequence and a tensor of a random seed. In contrast, the edge features include distances, movement vectors, shortest paths and rotation quaternions.

### Ablation study

**Figure 2:**
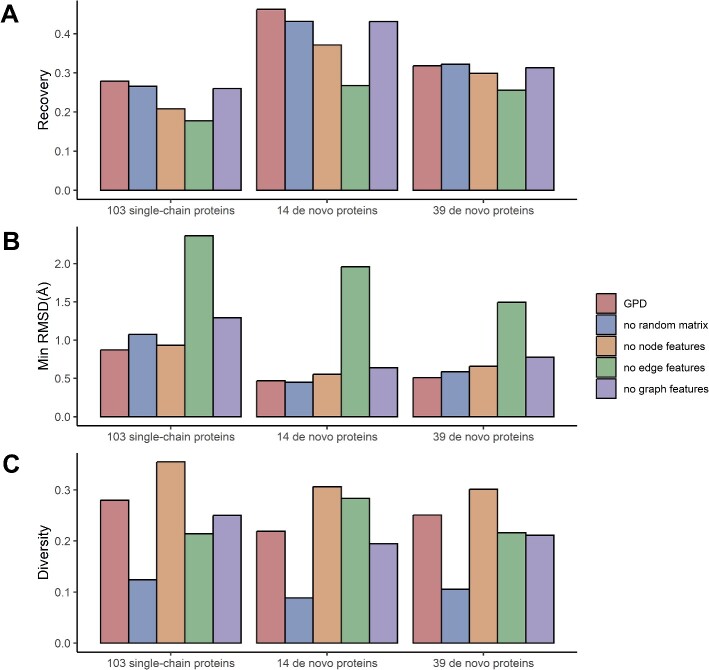
The recovery, RMSD and diversity with the different modules of the GPD model. (**A**) The recovery with the different modules of the GPD model on three test datasets. No random matrix ablated the random matrix inputted to the model. No node features ablated all the node features, including secondary classification, dihedral angle and the centrality of each residue. No_edge features ablated all the edge features, including distance, movement vectors and rotation quaternions. This makes GPD into a traditional Transformer using only node features. No graph features ablated all the graph features, including node centrality degree and node shortest pathways. (**B**) The min RMSD with the different modules of the GPD model. (**C**) The diversity with the different modules of the GPD model.

We conducted ablation studies to assess the impact of node features, edge features, graph features and a normally distributed random matrix on our model’s performance (Figure 2). The results show that the node features, edge features and graph features are essential for model’s performance. Interestingly, the model lacking node features still demonstrates relatively good performance compared with the model without edge model. This suggests that the edge feature might contain more information of the protein structure than the node feature. Incorporating a normally distributed random matrix can significantly enhance the diversity of designed sequences while maintaining comparable sequence recovery and folding capabilities.

### Performance of GPD model

The GPD model was trained using the CATH 40% sequential non-redundancy dataset, with a split ratio of 29 868:1000:103 for the training, validation and testing sets, respectively. We further evaluated the performance of GPD using 39 *de novo* proteins and 14 *de novo* proteins that exhibit significant structural differences from proteins belonging to natural folds [23, 24].

A comprehensive performance comparison was conducted between the GPD model and widely adopted design approaches, such as ProteinSolver, Structure Transformer, ESM-IF1 and ProteinMPNN. Four evaluation criteria, namely, recovery, diversity, pLDDT and root mean square deviation (RMSD), were employed to systematically assess the performance of these methods at both sequence and structural levels.

#### The performance of sequence diversity and recovery

Recovery and diversity are two crucial metrics in fixed-backbone sequence design, and they exhibit an interdependent relationship. Higher recovery often compromises sequence diversity, and conversely, increased diversity can reduce recovery. Consequently, it is essential to concurrently consider both recovery and diversity concurrently. It is generally accepted that two proteins with a sequence recovery exceeding 35% are likely to exhibit similar structures and perform analogous functions [25]. For *de novo* proteins, the recovery exceeds 30% for all five methods (except ProteinSolver), underscoring the need to enhance the diversity of designed sequences (Table 1).

**Table 1:** The performance of different methods

Protein classification	Methods	Training dataset	Recovery	Diversity	Min RMSD (Å)	Time^a^ (design 10 000 sequences)
103 single-chain proteins	ProteinSolver	Uniparc	0.191	0.526	0.984	5 h
	Structure Transformer	CATH	0.264	0.130	0.948	0.69 h
	ESM-IF1	CATH + AlphaFold2	0.261	**0.486**	0.703	55 h
	ProteinMPNN	CATH	0.260	0.237	**0.531**	3.11 h
	GPD	CATH	**0.279**	0.280	0.872	0.97 h
14 *de novo* proteins	ProteinSolver	Uniparc	0.246	0.498	0.557	/
	Structure Transformer	CATH	0.433	0.071	0.471	/
	ESM-IF1	CATH + AlphaFold2	0.363	**0.396**	0.374	/
	ProteinMPNN	CATH	**0.490**	0.165	**0.346**	/
	GPD	CATH	0.462	0.219	0.469	/
39 *de novo* proteins	ProteinSolver	Uniparc	0.217	0.487	0.768	/
	Structure Transformer	CATH	0.346	0.096	0.489	/
	ESM-IF1	CATH + AlphaFold2	0.336	**0.418**	0.476	/
	ProteinMPNN	CATH	**0.362**	0.177	**0.357**	/
	GPD	CATH	0.318	0.251	0.511	/

The best performance has been marked in bold. The best diversity except for ProteinSolver. ProteinSolver exhibited poor recovery across these datasets, and the high diversity compromises sequence recovery.

^a^Time: the task involves designing 10 000 sequences with 261 residues using a CPU.

As shown in Table 1, GPD achieved the highest recovery (27.9% ± 5.4%) for 103 single-chain proteins, while ProteinMPNN exhibited superior recovery for 14 *de novo* proteins (49.0% ± 8.3%) and 39 *de novo* proteins (36.2% ± 11%). Notably, ESM-IF1 outperformed in terms of diversity across all three test datasets, except for ProteinSolver. However, ProteinSolver exhibited low recovery (less than 30%) across these datasets and was hardly to use for protein design.

**Figure 3:**
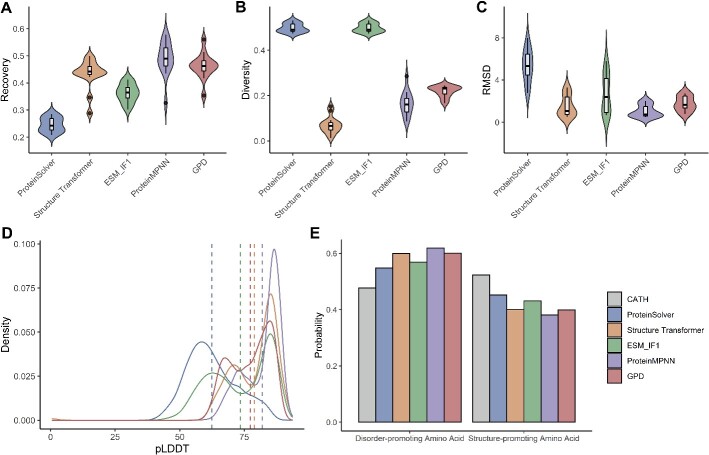
Comparison of designed sequence for five methods on 14 *de novo* proteins. (**A**) The sequence recovery between the designed sequences and the native sequence of the target structure. (**B**) The diversity of designed sequences. (**C**) RMSD for aligning the ESMFold predicted structures with the corresponding native structures. (**D**) The pLDDT scores of the ESMFold predicted structures. (**E**) The frequency of disorder-promoting amino acids (alanine, glycine, proline, arginine, glutamine, serine, glutamic acid and lysine) and structure-promoting amino acids (other 12 residues).

The time taken to design 10 000 sequences with 261 residues using a CPU was 55, 3.11 and 0.97 h for ESM-IF1 [17], ProteinMPNN [18] and GPD, respectively. The time consumption of ESM-IF1 was about 2.3 days, rendering ESM-IF1 unsuitable for high-throughput protein design. It is essential to note that our comparison just focused on high-throughput protein design models: Structure Transformer, ProteinMPNN and GPD. For the GPD model, the average recovery between the designed sequences and their corresponding native sequences was 46.2% ± 5.1% for 14 *de novo* proteins (Figure 3A), 31.8% ± 5.8% for 39 *de novo* proteins (Supplementary Figure S2) and 27.9% ± 5.4% for 103 single-chain proteins (Supplementary Figure S3). The average diversity among the designed sequences was 21.9% ± 2.4% (Figure 3B), 25.1% ± 3.3% (Supplementary Figure S2) and 28% ± 5.6% (Supplementary Figure S3). The GPD model exhibited significantly higher recovery and diversity compared to both Structure Transformer and ProteinMPNN on 103 single-chain proteins (Wilcoxon signed-rank test, *P*-values < 0.05). Moreover, the diversity of the GPD model was higher than that of Structure Transformer and ProteinMPNN on 103 single-chain proteins, 14 *de novo* proteins and 39 *de novo* proteins (Wilcoxon signed-rank test, *P*-values < 0.05). For 14 *de novo* proteins and 39 *de novo* proteins, the sequence recovery achieved an acceptable level (>30%) for the GPD model as well as the other two models [25].

In summary, Structure Transformer, ProteinMPNN and GPD have attained sequence recoveries at acceptable levels, which emphasizes the importance of increasing the diversity of designed sequences to expand the sequence space landscape. Higher diversity signifies that the designed sequences possess more variability and cover a broader range of the sequence space landscape. The GPD model achieved higher diversity than the other two methods on three test datasets.

#### The performance of structure folding for designed sequence

We utilized each of the five methods to design 100 sequences for every protein. These comprised 10 300 designed sequences for 103 single-chain proteins and 5300 sequences for 53 *de novo* proteins. We employed ESMFold to predict the structures of these designed sequences. The mean RMSD between the ESMFold-predicted structures and the corresponding native structures, along with the density plots of the pLDDT scores, are shown in Figure 3C and D for 14 *de novo* proteins, Supplementary Figure S2 for 39 *de novo* proteins and Supplementary Figure S3 for 103 single-chain proteins. The minimum RMSD was 0.469, 0.511 and 0.872 Å for 14 *de novo* proteins (Figure 3C), 39 *de novo* proteins (Supplementary Figure S2) and 103 single-chain proteins (Supplementary Figure S3), respectively. This indicates that GPD could generate sequences with good folding ability. Concurrently, all methods exhibited superior performance on 14 *de novo* proteins (Table 1). The predicted structure with the minimum RMSD is illustrated in Supplementary Figure S4. Protein folding ability was an important metric for evaluating the performance of different methods. All of these methods could generate a native-like folding structure.

Amino acids can be categorized into two groups based on their folding abilities: disorder-promoting amino acids (such as alanine, glycine, proline, arginine, glutamine, serine, glutamic acid and lysine) and structure-promoting amino acids (the remaining 12 residues). The presence of more structure-promoting amino acids could facilitate protein folding. All models designed sequences tended to have more disorder-promoting amino acids than folded protein in CATH dataset (Figure 3E). All proteins in CATH dataset were folded protein structures; however, the highest percentage of designed sequences with RMSD less than 2 Å (ProteinMPNN) was only 23.6% for single-chain proteins and the rate of foldable sequences in the experimental validation (50% for ProteinMPNN) was low. This suggests a limitation in the protein folding ability of the designed sequence, highlighting the necessity for filtering designed sequences.

### The performance of amino acids frequency for designed sequence

**Figure 4:**
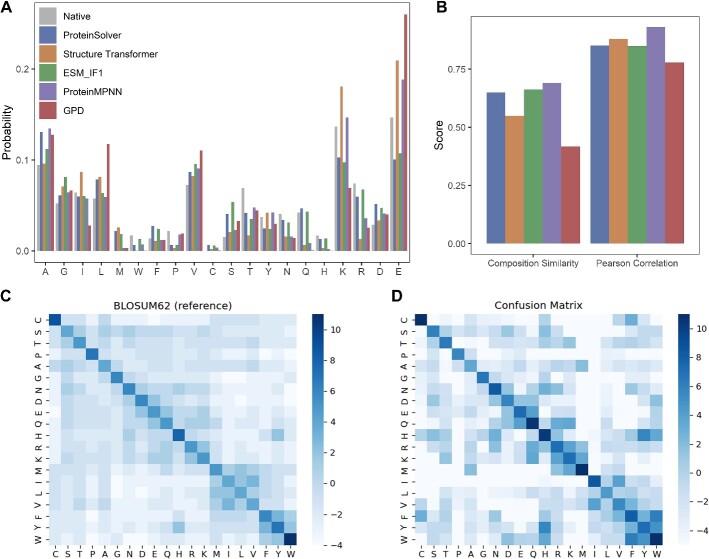
The amino acids frequency of designed sequence on 14 *de novo*. (**A**) The sequence identity between the designed sequence and the native sequence of the target structure. (**B**) The Pearson correlation coefficient and the composition similarity of the amino acid type compositions of the designed and the native sequences. (**C**, **D**) Confusion matrix between native sequence and design sequences, compared to BLOSUM62 as reference.

The frequency distributions of amino acid types for sequences designed using different methods, as well as for native sequences, are shown in Figure 4A for the 14 *de novo* proteins and in Supplementary Figure S5 for the 39 *de novo* proteins and 103 single-chain proteins. The Pearson correlation coefficient for the amino acid–type compositions of the designed and native sequences was 0.78, 0.80 and 0.81 for the 14 *de novo* proteins (Figure 4B), 39 *de novo* proteins (Supplementary Figure S5) and single-chain proteins (Supplementary Figure S5), respectively. The composition similarity was 0.42, 0.51 and 0.48. ProteinMPNN achieved the highest correlation (0.93) for the 14 *de novo* proteins, while ESM-IF1 obtained the highest correlation for the 39 *de novo* proteins (0.91) and single-chain proteins (0.97).

Certain side-chain types such as alanine, glutamic acid, leucine and valine had been utilized more frequently in the designed sequences than in the native sequences. In contrast, the use of side-chain types such as isoleucine, threonine, asparagine, glutamine and arginine significantly reduced in the designed sequences. All methods’ designed sequences exhibited a higher frequency of non-polar amino acids and a lower frequency of polar amino acids.

We calculated the substitution scores between the native sequences and the designed sequences using the same log-odds ratio formula as in the BLOSUM62 substitution matrix (Figure 4C and D). In the confusion matrices, the diagonal elements correspond to the largest substitution scores for all amino acids, suggesting that most amino acids in the designed sequences are physicochemical similar to their native counterparts. For instance, phenylalanine, tryptophan and tyrosine and lysine, arginine and methionine were similar in their respective pairs.

### Experiment validation

Only a few studies to date have reported experimental evaluations of protein sequences designed using deep learning. 3DCNN [15], ABACUS-R [16] and ProteinMPNN [18] have experimentally solved atomic structures for their designed sequences. ProteinSolver [13] and ProDESIGN-LE [21] have shown their designed sequences to have desired secondary structure contents and exhibit cooperative folding according to circular dichroism signatures. However, none of these methods analyzed the function and activity of the designed proteins.

In this study, we evaluated the activity of Candida antarctica lipase B (CalB) enzyme sequences designed by the GPD using wet-lab experiments. CalB was chosen for evaluation of the GPD model due to its remarkable tolerance to organic solvents and thermal stability, making it one of the most commonly employed industrial enzymes for hydrolytic reactions in biocatalytic applications [26]. CalB belongs to the $\mathrm{\alpha}$/$\mathrm{\beta}$ hydrolase family. Composed of 317 amino acids, CalB has a total structural weight of 33.46 kDa. The CalB structure was extracted from the Protein DataBank (PDB code: 1TCA) [27]. The substrate used in this study is *p*-nitrophenyl acetate (C2).

#### CalB design

CalB features a catalytic triad formed by residues S105, D187 and H224. The active-site cavity is tunnel-shaped, which constrains the steric positioning of substrates. In our design process, we kept 62 residue positions fixed. This included 5 active-site amino acids, 19 substrate pocket amino acids, 20 conserved sites from CalB single-site saturation mutagenesis data and 18 conserved sites from multiple sequence alignment (see Materials and methods for more details). Using the GPD model, we generated 1 million *de novo* designed sequences for CalB.

#### Functional screening

**Figure 5:**
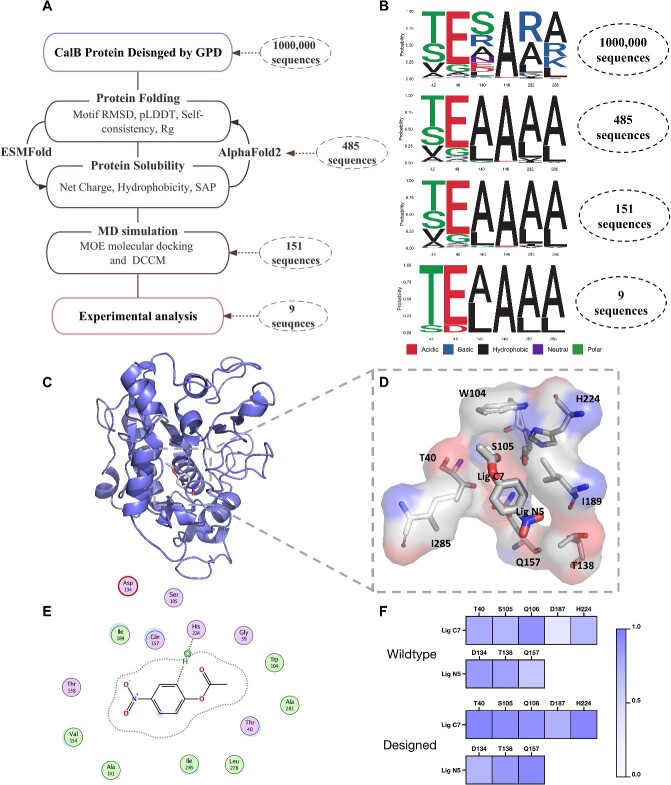
The design workflow of CalB hydrolase. (**A**) The design workflow of CalB including CalB sequences design, protein folding ability, protein solubility and MD simulation. (**B**) The seqlog plot of Thr42, Gln46, Leu140, Ala146, Ala282 and Val286 after each step screening. (**C**) The CalB and substrate (*p*-nitrophenyl acetate C2) complex after MD simulation. (**D**) The region of CalB active sites is enlarged. (**E**) The 2D interaction diagram between CalB and substrate. (**F**) The dynamic cross-correlation matrix (DCCM) characterizes the significant interactions of CalB active sites and substrate.

The functional screening of 1 million CalB-designed sequences is shown in Figure 5A. The designed sequences were virtually screened on the basis of protein folding ability, protein solubility and molecular dynamics (MD) simulation.

##### Protein folding ability

We implemented ESMFold and AlphaFold2 to predict the structures of the proposed 1 million sequence designs. To evaluate the folding capability of these designs, several parameters were used: the RMSD of 62 conserved sites between the forecasted structures and native CalB structures, the predicted local distance difference test (pLDDT) scores, consistency between ESMFold and AlphaFold2 predictions and the radius of gyration (Rg) comparing the predicted structures with the native CalB structures.

##### Protein solubility

To estimate protein solubility, we took into account the net charge, hydrophobicity and spatial aggregation propensity (SAP) score. Following an assessment of protein folding and solubility, 151 sequences were selected from the initial design set.

##### MD simulations

MD simulations were carried out for the 151 protein–ligand complexes derived from Molecular Operating Environment (MOE) docking results. Following these simulations, nine sequences, which align with the catalytic mechanism, were selected for experimental validation. The RMSD of these sequences varied between 2.29 and 3.38 Å, with recovery rates ranging from 0.445 to 0.498. The diversity among these nine sequences was found between 0.215 and 0.253.

Comprehensive data related to each step of the screening process are listed in Supplementary Table S1. Figure 5B illustrates the active sites of the CalB enzyme, while Figure 5C and Supplementary Figure S6 display a seqlog plot of the key residues post-screening. This indicates our virtual screening workflow’s effectiveness in selecting residues that align with the required chemical properties. The residues of Thr42, Gln46, Leu140, Ala146, Ala282 and Val286 were identified in proximity to the conserved sites.

#### Experimental validation

**Figure 6:**
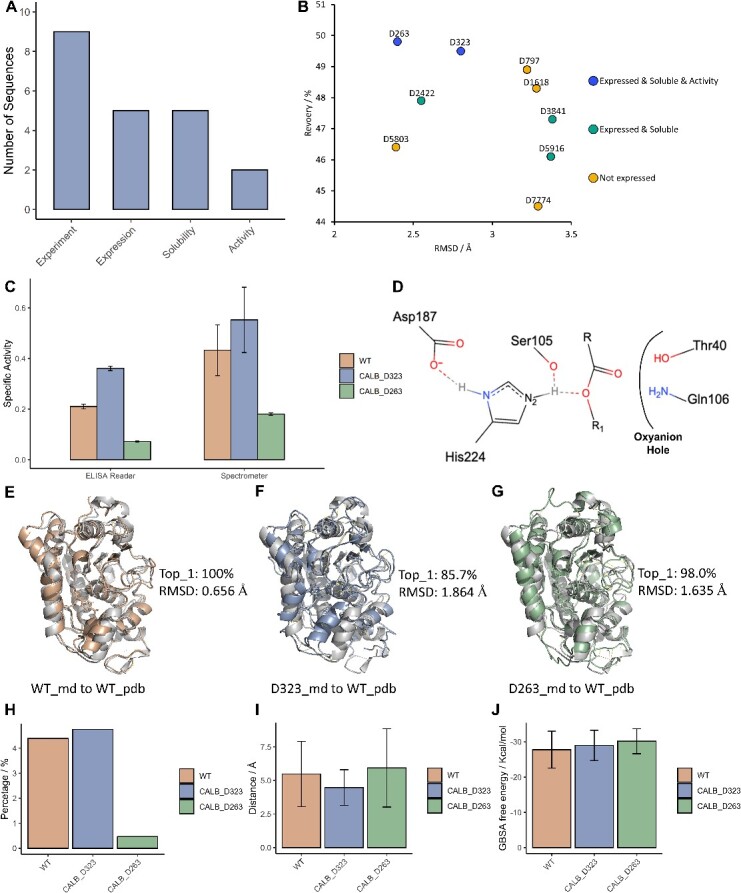
Experiments and MD simulation results of designed sequences. (**A**) The number of designed sequences for experimented and expressed. (**B**) The RMSD and recovery values of designed sequences. (**C**) The specific activity of designed sequences and wild type using enzyme linked immunosorbent assay (ELISA) Reader. (**D**) The model of pre-catalytic state of CALB-substrate complex. (**E**) The alignment between MD simulation clustered wild type (WT) structure and WT pdb structure. (**F**) The alignment between MD simulation clustered D323 structure and WT pdb structure. (**G**) The alignment between MD simulation clustered D263 structure and WT pdb structure. (**H**) The hydrogen bond percentage during the MD simulation trajectories. (**I**) The distance between Ser105 side-chain gamma oxygen atom and the carbonyl carbon atom. (**J**) The GBSA binding free energy.

Nine sequences that fulfilled the catalytic mechanism were chosen for experimental validation (Supplementary Figure S7). Out of these nine designed sequences, five yielded successful protein expression in yeast. All expressed proteins were soluble post-purification. Notably, two designed sequences, D263 and D323, demonstrated catalytic activity (Figure 6A and B). The success rate underscores the effectiveness of our design and screening methodology.

As shown in Figure 6B, the designed proteins D263 and D323 not only showed catalytic activity but also exhibited lower RMSD and higher recovery compared to their inactive counterparts. This outcome indicates the potential usefulness of our screening workflow.

The purified proteins underwent specific activity analysis using an ELISA Reader. The specific activity, measured by the ELISA Reader, was 0.210 ± 0.0065, 0.361 ± 0.0089 and 0.072 ± 0.0029 U/mg for the CalB native sequence, D323 and D263, respectively. Of these, D323 had a higher hydrolytic activity than that of the CalB native sequence (*P*-value is 0.029). The experimental results demonstrate that the *de novo* CalB design sequence had higher activity than the CalB native sequence. We further evaluated the substrate selectivity of the designed sequences. As shown in Supplementary Figure S8, the designed sequences have strong selectivity on six substrates (C2, C4, C6, C8, C12 and C16) and C2 is the most favorable substrate because our sequence screening is based on C2.

Further insight into the experimental results was gained through MD simulations conducted for the wild type, D323 and D263. For each system, three parallel trajectories of 200 ns were run. Figure 6E–G revealed that the designed proteins had higher RMSD compared to the wild-type crystal structure. Yet, the RMSD of the simulated structure was considerably lower than the AlphaFold2 predicted one (shown in Figure 6B), suggesting that MD simulation could enhance structure prediction results. Figure 6D illustrates that the hydrogen bond between Ser105 and His224 is vital to CALB’s catalytic capacity. Figure 6H demonstrates that the wild type and D323 protein exhibit a higher percentage of hydrogen bonds compared to D263. This observation could partly explain the experimental results. Additionally, the distance between the Ser105 side-chain gamma oxygen atom and the ligand carbonyl carbon atom of D323 was lower than that of the wild type and D263, as shown in Figure 6I. This finding might explain why D323 had the highest catalytic ability among the three. Nevertheless, the generalized born surface area (GBSA) results in Figure 6J show no significant difference among the three systems, suggesting that GBSA results might only indicate binding affinity, not catalytic capability.

### GPDGenerator Webserver

As illustrated in Supplementary Figure S9, we have developed and made publicly accessible a user-friendly online tool, the GPDGenerator (https://yu.life.sjtu.edu.cn/ChenLab/GPDGenerator/). The server utilized parameters derived from our trained model to design protein sequences based on the input PDB file.

The ‘Introduction’ interface provided an overview of GPD and showcases examples of GPD results. The ‘Analysis’ interface allowed users to input a PDB file and design specific amino acids at specified positions. The ‘Results’ interface output the designed sequences in FASTA format and provided the recovery along with the corresponding native sequence.

It’s important to note that the protein length should not exceed 400 amino acids, and the number of designed sequences should be kept under 100 to conserve computational resources. In conclusion, this online tool was designed to facilitate the generation of novel protein sequences based on a fixed backbone.

## DISCUSSION

This study introduces a graph representation–based Transformer, GPD, designed to tackle fixed-backbone protein sequence design. When compared to existing deep learning protein sequence design methodologies, the primary contributions of our GPD model are 2-fold. Firstly, the GPD model integrates five graph node encodings and four edge encodings, harnessing the protein’s spatial information effectively. A normally distributed random matrix was also incorporated into node features to augment the diversity of the designed sequences. Secondly, functional filtering, based on structure folding, solubility and function, was performed to boost the experimental success rate. The GPD-designed sequences of CalB hydrolase demonstrated higher specific activity than the CalB wild type.

The performance of time efficiency for GPD model is also predominant. For instance, the time taken to design 100 sequences with 261 residues using a CPU were 180, 25, 1980, 112, 540 000, 247 100 and 35 s for ProteinSolver [13], Structure Transformer [10], ESM-IF1 [17], ProteinMPNN [18], 3D CNN [15], ABACUS-R [16] and GPD, respectively. Compared to the three methods (3DCNN, ABACUS-R and ProteinMPNN) with experimental validations by crystallography, the GPD model required less time which is better for high-throughput sequence generation.

The GPD model has achieved higher recovery and diversity across various proteins types. Existing methods have primarily focused on improving protein sequence recovery. However, the native recovery rate should not be considered as the ‘gold standard’ for benchmarking different methods [28]. The sequence space is vast due to the potential combinations of 20 amino acid residues. Ideally, the designed sequences should cover a wide range of this sequence space landscape, exhibiting high diversity [16]. In functional protein sequence design challenges, an increased diversity in models can significantly broaden the range of sequence options available for subsequent functional assays. This expanded pool of options improves the likelihood of identifying sequences that truly possess the desired functionality, as evidenced by our high functional sequence rate (two out of nine).

High recovery alone is not an adequate metric for predicting the performance of design methods in wet laboratory experiments [16, 18, 29, 30]. High recovery has not shown strong correlations with the success rate of wet experiments, since a single residue substitution, which may not cause notable changes in metrics, can nevertheless disrupt the overall structure [31]. The capability to express and purify designed proteins is crucial the success of wet experiments. Functional filtering, based on structure folding, solubility and function, is key to improving the success rate of wet experiments [32]. Assessing structure folding and solubility computationally provides useful protocols for evaluating the design sequences.

In this study, we used GPD to design 1 million *de novo* sequences of CalB hydrolase. Nine sequences post functional filtering were examined by wet experiments. Over half of the designed sequences, specifically five out of nine, were successfully expressed and purified. Two out of the nine experimentally evaluated designed sequences of CalB exhibited hydrolytic activity. Notably, the specific activity of one designed sequence (0.36) was significantly higher than that of the CalB wild type (0.21). Furthermore, the designed sequences have strong substrate selectivity on six substrates with different carbon chain lengths (C2–C16). The high success rate of GPD’s experimental design, coupled with computational efficiency and no requirement for customization, makes GPD highly useful for protein design.

Despite the satisfactory performance of our method on fixed-backbone protein sequence design, there are opportunities for further enhancement. Firstly, the protein folding capability of the designed sequence is limited. Although the minimum RMSD for these models is less than 1.0 Å, only 23.6% of designed sequences achieve an RMSD under 2 Å for single-chain proteins (ProteinMPNN). All methods still suffer from protein folding deficiencies, leading to experimental failure. Thus, the protein folding ability is a crucial metric to evaluate the performance of different methods. Secondly, the number of expressed and soluble proteins is not particularly high compared to ProteinMPNN and ABACUS-R. The ratios of expressed and soluble proteins are 76% and 86% for ProteinMPNN [18] and ABACUS-R [16], respectively, while for GPD, the ratio is only 56%. Lastly, the fixed-backbone sequence design for different types of proteins should be validated by wet experiments in future studies.

## MATERIALS AND METHODS

### Feature representation

In order to obtain as much structure information as possible and satisfy the SE [3] equivariance, we treated each single protein main-chain structure as a graph that contains both node features and edge features. We took every single residue as a node and took the connection between residues as the edge of the graph. All node features and edge features could be calculated by only the backbone atom information and satisfied the SE [3] equivariance.

The node features contained the main-chain dihedral angles $psi$ and $phi$, the secondary classification, the centrality of each residue, the pre-designed protein sequence and a tensor of random seed. For the main-chain dihedral angle, both $phi$ and $psi$ were embedded in the ways of sine and cosine function (shown in Figure 1A). This information redundancy could help the neural network better learned the features. We used the Define Secondary Structure of Proteins (DSSP) algorithm to classify the main-chain secondary structure. Eight class of secondary structures were used in this study, such as ${3}_{10}$-helix, $\alpha$-helix, $\pi$-helix, hydrogen bonded turn, $\beta$-harpin, $\beta$-bridge, bend and loop. The centrality of a single residue was represented by the betweenness centrality and calculated by


(1) \begin{equation*} {C}_b(k)=\sum_{i\ne j\ne k,i<j}\frac{g_{ij}(k)}{g_{ij}}. \end{equation*}

where ${c}_b(k)$ means the betweenness centrality of node $k$. ${g}_{ij}$ means the number of shortest paths that start from node $i$ then end with node $j$, and at the same time pass node ${v}_i$. ${g}_{ij}$ means the number of all shortest paths between node $i$ and $j$.

We have leaved an API for pre-designed sequence embedding. The user could pre-design each residue at each position and the other residues will be generated according to both the structure information and the pre-designed sequence. If there is no pre-designed residue requirement, the pre-designed sequence tensor would be set as all-zeros. Taking Figure 1B for example, when redesigning GFP, we could predefine the chromophoric residues that could not be predicted by the backbone structure but necessary for GFP function (citation for GFP).

The random tensor was used for enlarging the designed sequence space, which is of essential importance of *de novo* protein design. Our goal was to get a higher level of neural network randomicity when training at the same level of loss value.

The edge features contained the distances, the movement vectors, the shortest pathway and the rotation quaternions. For the distances map, we calculated the distances between the alpha carbon atoms of each residue (shown in Figure 1D). For the movement vector, we used the coordinate system transformation to satisfy the SE [3] equivariance. First, by using the residue gas shown in Figure 1D, (citation for residue gas) we defined a residual specific coordinate system $O$, defined as


(2) \begin{equation*} O=\left[\overrightarrow{C_{\alpha }C},\overrightarrow{C_{\alpha }N},\overrightarrow{C_{\alpha }C}\times \overrightarrow{C_{\alpha }N}\right]. \end{equation*}

The residual specific coordinate system based on movement vector ${v}_m$ was calculated by


(3) \begin{equation*} {v}_m={O}^T\frac{\overrightarrow{C_{\alpha }{C}_{\alpha}^{\prime }}}{\parallel{\overrightarrow{C}}_{\alpha }{C}_{\alpha}^{\prime}\parallel }. \end{equation*}

where ${v}_m$ means the transferred movement vector, $\overrightarrow{C_{\alpha }{C}_{\alpha}^{\prime }}$ means the initial movement vector under the Cartesian coordinate system.

For the rotation relation between two residues, we used rotation quaternion for representation, defined as


(4) \begin{equation*} {q}_{i,j}=q\left({O}_i^T{O}_j\right). \end{equation*}

where ${q}_{i,j}$ means the quaternion number, $q\left(\cdotp \right)$ means the operation that transfer the rotation matrix into rotation quaternion number. The ${O}_i$ and ${O}_j$ are the two residual coordinate system representations of residue $i$ and $j$, respectively.

The shortest pathway feature was calculated based on the distance matrix, also a way of feature redundancy. We set the cut of 12 Å between two carbon alpha atoms to define whether there is a contact between two residues. By using this contact map, we calculated the length of the shortest path between each residue pairs, shown in Figure 1E for an example.

### Model structure

The overall architecture of GPD is shown in Figure 1A, using only the encoder part of the real Graphormer for save more computational resources. Here, we took the whole protein backbone as an embedded graph $\mathcal{G}=\left(\mathcal{V},\mathcal{E}\right)$ where $\mathcal{V}=\left\{{\mathcal{v}}_1,\dots, {\mathcal{v}}_{\mathrm{N}}\right\}$ describe the residual level of node features ($\mathrm{N}$ is the protein sequence length) and $\mathcal{E}=\left\{{\mathrm{e}}_{\mathrm{ij}}\right\}$ describe the edge features ($\mathrm{i}$ and $\mathrm{j}$ refer to the residue index). The detailed feature embedding processes are shown in section Feature representation. Six recycles of the Graphormer attention block were used. The key component of Graphormer was the Graphormer block and is shown in Figure 1B. The main difference between Graphormer and traditional Transformer was the adding of the embedded edge feature. In every Graphormer block, the interaction weight between two residues’ node features was determined by both the attention matrix and the edge feature matrix. This allowed the structural information to flow from edges to nodes. Also, the node information could flow to the edges in the second Graphormer block.

For each head of the Graphormer multi-head attention block, the layer update process was as follows. Let $\mathcal{H}=\left\{{\mathcal{h}}_1,\dots, {\mathcal{h}}_{\mathrm{N}}\right\}\in{\mathbb{R}}^{\mathrm{N}\times \mathrm{d}}$ be the embedded node feature, where $\mathrm{N}$ is the dimension of protein sequence and $\mathrm{d}$ is the hidden dimension. ${\mathcal{h}}_{\mathrm{i}}$ is the hidden representation of residue $\mathrm{i}$. The input $\mathcal{H}$ s projected by three matrices ${\mathrm{W}}_{\mathrm{Q}}\in{\mathbb{R}}^{\mathrm{d}\times{\mathrm{d}}_{\mathrm{K}}}$, ${\mathrm{W}}_{\mathrm{K}}\in{\mathbb{R}}^{\mathrm{d}\times{\mathrm{d}}_{\mathrm{K}}}$ and ${\mathrm{W}}_{\mathrm{V}}\in{\mathbb{R}}^{\mathrm{d}\times{\mathrm{d}}_{\mathrm{V}}}$ to the corresponding $\mathrm{Q}$, $\mathrm{K}$ and $\mathrm{V}$. Let ${\mathcal{H}}_{\mathrm{e}}=\left\{{\mathcal{h}}_{\mathrm{e}\left(\mathrm{i},\mathrm{j}\right)}\right\}\in{\mathbb{R}}^{\mathrm{N}\times \mathrm{N}\times{\mathrm{d}}_{\mathrm{e}}}$ where ${\mathcal{h}}_{\mathrm{e}\left(\mathrm{i},\mathrm{j}\right)}$ is the embedded edge feature representation between residue $\mathrm{i}$ and $\mathrm{j}$ and ${\mathrm{d}}_{\mathrm{e}}$ is the hidden dimension. The Graphormer self-attention weight ${A}_{ij}$ could be calculated as


(5) \begin{equation*} {A}_{ij}=\frac{\left({\mathcal{h}}_i{W}_Q\right){\left({\mathcal{h}}_j{W}_K\right)}^T}{\sqrt{d_K}}+{C}_{ij} \end{equation*}

where ${C}_{ij}$ could be calculated as follows:


(6) \begin{equation*} {C}_{ij}=\frac{1}{d_e} \end{equation*}

where $n$-th dimension of the edge feature between residue $\mathrm{i}$ and $\mathrm{j}$. The output of the single self-attention layer could be calculated as


(7) \begin{equation*} \mathrm{Attn}\left(\mathcal{H}\right)=\mathrm{softmax}\left(\mathrm{A}\right)\mathrm{V},\mathrm{V}=\mathcal{H}{\mathrm{W}}_{\mathrm{V}} \end{equation*}

The embedding blocks for edge features and node features are shown in Figure 1C and D, respectively. The Distances map, the movement vector matrix, the rotate quaternion matrix and the shortest path number matrix were all joined at the last dimension and passed through two layers of fully connected neural networks. The last dimension of the edge features matrix was transferred into the exact dimension of the number of the attention heads in the multi-head attention block.

We utilized the layer normalization (LN) before the multi-head self-attention (MHA) and the feed-forward blocks (FFN). The number of head is 10 and the dimension of feed-forward is 1024. The designed sequences were generated by passing through a linear layer followed by the softmax operation. The cross-entropy loss was computed between the predicted amino acid types and the original ones. Let $L\left(x,y\right)$ be the loss value,


(8) \begin{equation*} L\left(x,y\right)=\frac{\sum_n^N{l}_n}{N} \end{equation*}

where *x* is the input and *y* is the output, *N* is the number of the amino acid residues of a single protein sequence. The ${l}_n$ could be calculated as follows:


(9) \begin{equation*} {l}_n=\mathit{\log}\frac{\sum_{c=1}^C\mathit{\exp}\left({x}_{n,c}\right)}{\mathit{\exp}\left({x}_{n,{y}_n}\right)} \end{equation*}

where *C* = 22, which represents the residue types during the training processes (20 amino acid types, 1 unknown type and 1 padding type). We used Adam as the optimizer with a batch size of 64, and the learning rate was set to 0.002. GPD was trained on 1 NVIDIA 40G A100 GPUs for approximately 1 day. GPD took 35 s to design 100 sequences with 261 residues using a CPU. GPD demonstrates high efficiency in protein sequence design.

### Data set and benchmark metrics

We used the CATH 40% sequential non-redundancy dataset for neural network training, validation and testing. The split ratio between training, validating and testing set was 29 868:1000:103. We trained the network for 400 epochs with the randomly masked pre-designed sequence and validated with the fully masked pre-designed sequence. Fourteen *de novo* proteins, 39 *de novo* proteins and 103 single-chains proteins were used to evaluate the performance of GPD model and other existed methods.

Recovery was the proportion of the same amino acids at equivalent position between the native sequence and the designed sequence, calculated with Equation (10). Diversity was one minus the proportion of the same or similar amino acids at equivalent position between designed sequence, calculated with Equation (11). RMSD quantifies the differences between the predicted structures and the corresponding native structures. RMSD was calculated as Equation (12).


(10) \begin{align*} &\kern-.6pc Recovery=\nonumber\\&\kern-.6pc\frac{\#\ of\kern0.17em same\kern0.17em amino\kern0.17em acids\kern0.17em between\kern0.17em native\kern0.17em and\kern0.17em designed\kern0.17em seuqnce}{the\kern0.17em length\kern0.17em of\kern0.17em sequence}\ast 100. \end{align*}

(11) \begin{align*}&\kern-.6pc Diversity=\nonumber\\&\kern-.6pc1-\frac{\#\ of\kern0.17em same\kern0.17em or\kern0.17em similar\kern0.17em amino\kern0.17em acids\kern0.17em between\kern0.17em designed\kern0.17em seuqnce}{the\kern0.17em length\kern0.17em of\kern0.17em sequence}\ast 100. \end{align*}

(12) \begin{equation*} RMSD=\sqrt{\frac{1}{N}\sum_{i=1}^N{\left({r}_i^p-{r}_i^n\right)}^2}. \end{equation*}

where ${r}_i^p$ and ${r}_i^n$ are Cartesian coordinates of the *i*-th atom from predicted structure ${r}^p$ and the native structure ${r}^n$, respectively. *N* is the number of atoms.

### CalB design

Sixty-two residue positions were fixed according to the enzyme catalytic mechanism. Residues S105, D187 and H224 were catalytic triad; T40 and Q106 were oxyanion hole; A141, L144, V149 and I285 were substrate hydrophobic pocket; and D134, T138 and Q157 were substrate hydrophilic pocket. Twenty conserved residues (38, 39, 107, 108, 109, 110, 111, 133, 180, 181, 182, 190, 209, 230, 79, 130, 131, 132, 135, 228) were from CalB single site-saturation mutagenesis data. Eighteen conserved residues (103, 104, 190, 69, 180, 209, 216, 239, 188, 258, 150, 136, 294, 127, 74, 98, 169, 64) were from multiple sequence alignment. MD simulation shows that 15 residues (268–280, 225, 154) were important to keep the steric positioning of substrates. These 62 conserved residues of CalB were fixed upon using GPD to design 1 million *s*equences.

### Functional screening

The designed sequences were virtually screened based on protein folding ability, protein solubility and MD simulation. According to the structure of CalB, seven residues (277, 280, 281, 285, 139, 188, 38) near substrate hydrophobic pocket should be non-polar. 40 278 designed sequences were non-polar amino acid on these seven residues. ESMFold was used to predict the structure of these sequences. The detailed information is listed in Supplementary Table S1. Firstly, we applied ESMFold to predict the structures of the 40 278 designed sequences. 485 sequences were met the screening criteria of protein folding ability and protein solubility. Secondly, we used AlphaFold2 to predict the structures of these 485 sequences; 151 sequences were filtered according to protein folding ability and protein solubility. MD simulations were carried out for the 151 protein–ligand complexes. Nine sequences met the catalytic mechanism and were chosen for experimental validation.

#### Protein folding ability

The radius of gyration (Rg) of ${C}_{\alpha }$, the RMSD of 62 conserved sites [Equation (12)] and the pLDDT scores were used to estimate the folding ability of designed sequences. Rg of ${C}_{\alpha }$ determines the compactness of predicted structures; smaller means that the protein structure is more compactness and stable. The Rg was calculated by mdtraj [23, 33, 34], and the Rg of designed proteins should be less than that of CalB native structure (18.45 Å). RMSD quantifies the differences of 62 conserved sites between the predicted structures and the CalB native structure. The RMSD of 62 conserved sites should be less than 1.5 Å, and the pLDDT of predicted structures should be more than 80. The Rg only measures the folding ability of predicted proteins as a whole, while the RMSD of 62 conserved sites guarantees the similarity of activity sites.

#### Protein solubility

The net charge, hydrophobicity and SAP were used to estimate the protein solubility. The net charge of a protein was important for its solubility; neutral or positively charged proteins were more likely to lead to aggregation, and neutral or positively charged proteins might have non-specific binding with negatively charged DNA (Equation (13)). SAP was calculated for each residue by a combination of solvent accessibility area and hydrophobicity, calculated by Rosetta. Hydrophobicity controls the non-polar residues on the surface (Equations (14) and (15)) [31].


(13) \begin{equation*} \mathrm{Net}\ \mathrm{change}=\# Arg+\#\mathrm{Lys}-\# Asp-\# Glu \end{equation*}

(14) \begin{equation*} {n}_i=\sum_{j=1}^L1/\left(1+\exp \left({d}_{ij}-m\right)\right)\ast{\left(\left(\cos \left(\pi -{\phi}_{ij}\right)+a\right)/\left(1+a\right)\right)}^b \end{equation*}

(15) \begin{align*} \mathrm{Hydrophobicity}=&\sum_{i=1}^L{\delta}_i^{\ast}\left[1-\mathrm{sigmoid}\left({n}_i-{n}_0\right)\right]\Big/\nonumber\\&\sum_{i=1}^L\left[1-\mathrm{sigmoid}\left({n}_i-{n}_0\right)\right] \end{align*}

where ${d}_{ij}$ and ${\phi}_{ij}$ are the ${C}_b-{C}_b$ distance and $Ca- Cb/ Ca- Cb$ angles between residues *i* and *j* and *m* = 1, *a* = 0.5 and *b* = 2 are tuning parameters set to their default values. ${n}_0$ was the median of ${n}_i$. ${\delta}_i^{\ast }$ = 1 if residue *i* is non-polar (V, I, L, M, W, F) and 0 otherwise. The quantity $1-\mathrm{sigmoid}\left({n}_i-{n}_0\right)$ ranges from 0 to 1 and is higher when a residue is closer to the surface. More nonpolar residues on the surface would disrupt protein folding.

#### MD simulations

MD simulations were carried out for the 151 protein–ligand complexes from MOE docking results. Because CALB was an ordered protein with many short disordered regions, ff03CMAP force field was used for simulation [35]. This force field was developed by our group and was proved to balance the ordered–disordered region co-existing systems (Recent force field strategies for intrinsically disordered proteins). The solvent model used was TIP4P-Ew [36], a model proved to be suitable for ordered protein [37, 38]. The antechamber was used to parameterize the ligand molecule [39]. Firstly, energy minimization, heating and equilibrium of the system were carried out. The energy of the system was minimized by the steepest descent method of 3000 steps and the conjugate gradient method of 3000 steps. After energy minimization, the system was heated from 0 to 321 K in a time of 50 ps and then performs an energy balance of 100 ps at constant pressure and temperature of 321 K. In the whole process, the long-range electrostatic interaction was calculated by PME algorithm, and the covalent bonds of all hydrogen atoms were constrained by SHAKE algorithm. The cut-off value for the van der Waals interaction and the short-range electrostatic interaction was set at 8 Å. The final simulation process was carried out at NPT and temperature of 321 K, and the simulation time was 20 ns. Nine sequences with reasonable conformation were selected for experimental validation. After experiments, MD simulation of the wild-type complex, the D323 and the D263 were performed with three parallel trajectories for 200 ns.

#### Trajectory analysis

The dynamic cross-correlation matrix was calculated as follows (Equation (16)):


(16) \begin{equation*} {C}_{ij}=\varDelta \overrightarrow{R_i}\frac{\left\langle \varDelta \overrightarrow{R_i}\right.\left.\cdot \varDelta \overrightarrow{R_j}\right\rangle }{\sqrt{\left\langle{\left|\varDelta \overrightarrow{R_i}\right|}^2\right\rangle \left\langle{\left|\varDelta \overrightarrow{R_j}\right|}^2\right\rangle }} \end{equation*}

where ${C}_{ij}$ is the cross-correlation of atom $i$ and atom $j$, $\left\langle \right\rangle$ denotes time averaging and $\varDelta \overrightarrow{R_i}$ and $\varDelta \overrightarrow{R_j}$ represent the displacement of atom $i$ and $j$, respectively. When calculating DCCM, the python package MDtraj was used for trajectory loading [23, 34]. The cpptraj was used for RMSD, distance, hydrogen bond and structure cluster analysis. The DBSCAN method was used for clustering [40], taking 1 frame every 100 frames. MinPoints was set to 10, and epsilon was set to 3.0. When calculating the hydrogen bond, the distance cut-off was set to 0.3 nm, while the angle cut-off was set to 120°. The generalized Born surface area (GBSA) model was used to calculated the binding free energies of the protein–ligand complexes [41].

### Heterologous expression and purification of CalB

The *Escherichia coli* Rosetta (DE3) with the recombinant plasmid of pET22b-CalB designed sequences was cultivated for 3 h at 37°C in 2× yeast extract tryptone (2YT) with ampicillin (100 μg/ml) and chloramphenicol (34 μg/ml). Then, the final concentration of 0.1 mM IPTG (isopropylβ-D-thiogalactoside) was added and induced overnight at 15°C. Cells were collected by centrifugation at 5000 rpm for 10 min. The designed proteins were purified by Nickel column affinity chromatography. The purified proteins were detected by sodium dodecyl sulfate polyacrylamide gel electrophoresis (SDS-PAGE). The recombinant plasmid of pET22b-CalB was synthesized in GENEWIZ Company (Suzhou, China).

### Enzyme activity assays

The *p*-nitrophenyl acetate C2 was used to determine the activity of CalB designed sequences and CalB wild type. The ability of enzymatic hydrolysis was measured by ELISA. The reaction system consisted of *p*-nitrophenyl acetate C2 (200 M) and 100 L enzyme solution, and the reaction mixture was supplemented to 1 ml by 50 mM PBS (pH 7.5). The enzymatic reaction was carried out at 37°C for 5 min. One unit of enzymatic activity (U) was defined as the amount of enzyme required to hydrolyze the substrate to produce 1 μmol of *p*-nitrophenol per min. *p*-nitrophenyl acetate C2 (CAS No. 830-03-5) was from Sigma-Aldrich (St. Louis, MO, USA).

**Key Points**

- This is the first time to build Graphormer-based architecture for protein design (named GPD) to efficiently generate protein sequence.
- The performance of GPD is significantly better than that of state-of-the-art model for ProteinMPNN on multiple independent tests, especially for sequence diversity.
- GPD was successfully used to discovery CalB hydrolase with high catalytic activity and substrate selectivity.

## Supplementary Material

GPD_20240131_SI_re_bbae135

## Data Availability

The code for training GPD is available at https://github.com/decodermu/GPD. The structures generated in this article will be shared on reasonable request to the corresponding author.

## Author Biographies

**Junxi Mu** is an undergraduate student studying bioinformatics at Shanghai Jiao Tong University. His research direction is molecular dynamics simulations, enhanced sampling and deep learning.

**Zhengxin Li** is a PhD student studying bioinformatics at Shanghai Jiao Tong University. His research direction is molecular dynamics simulations and biological force field.

Bo Zhang is an undergraduate who is major in bioinformatics at Shanghai Jiao Tong University. His research direction is protein design via deep learning.

**Qi Zhang** is a PhD who is major in microbiology at Shanghai Jiao Tong University. Her research direction is enzyme express and purification.

**Jamshed Iqbal** is a professor who is major in chemistry at COMSATS University Islamabad. His research direction is innovation drug discovery.

**Abdul Wadood** is a professor who is major in bioinformatics at Abdul Wali Khan University Mardan. His research direction is molecular dynamics simulation.

**Ting Wei** is an assistant professor who is major in bioinformatics at Shanghai Jiao Tong University. Her research direction is protein design based on deep learning.

**Yan Feng** is a professor who is major in biochemistry at Shanghai Jiao Tong University. Her research direction is enzyme design and synthetic biology.

**Hai-Feng Chen** is a professor who is major in bioinformatics at Shanghai Jiao Tong University. His research direction is *de novo* protein design based on deep learning and innovation drug design.

## References

[1] Lu H , Diaz DJ, Czarnecki NJ, et al. Machine learning-aided engineering of hydrolases for PET depolymerization. Nature 2022;604:662–7. 35478237 10.1038/s41586-022-04599-z

[2] Cao L , Goreshnik I, Coventry B, et al. De novo design of picomolar SARS-CoV-2 miniprotein inhibitors. Science 2020;370:426–31. 32907861 10.1126/science.abd9909 PMC7857403

[3] Huang P-S , Boyken SE, Baker D. The coming of age of de novo protein design. Nature 2016;537:320–7. 27629638 10.1038/nature19946

[4] Defresne M , Barbe S, Schiex T. Protein design with deep learning. Int J Mol Sci 2021;22:11741. 34769173 10.3390/ijms222111741 PMC8584038

[5] Wang J . Protein sequence design by deep learning. Nat Comput Sci 2022;2:416–7. 38177862 10.1038/s43588-022-00274-5

[6] Wu Z , Johnston KE, Arnold FH, Yang KK. Protein sequence design with deep generative models. Curr Opin Chem Biol 2021;65:18–27. 34051682 10.1016/j.cbpa.2021.04.004

[7] Leaver-Fay A , Tyka M, Lewis SM, et al. ROSETTA3: An object-oriented software suite for the simulation and design of macromolecules. In: Methods in Enzymology, Vol. 487. Elsevier, 2011, 545–74. 21187238 10.1016/B978-0-12-381270-4.00019-6 PMC4083816

[8] Ding W , Nakai K, Gong H. Protein design via deep learning. Brief Bioinform 2022;23:bbac102. 35348602 10.1093/bib/bbac102 PMC9116377

[9] O’Connell J , Li Z, Hanson J, et al. SPIN2: predicting sequence profiles from protein structures using deep neural networks. Proteins 2018;86:629–33. 29508448 10.1002/prot.25489

[10] Ingraham J , Garg V, Barzilay R, Jaakkola T. Generative models for graph-based protein design. Adv Neural Inf Process Syst 2019;32:15820–31.

[11] Qi Y , Zhang JZ. DenseCPD: improving the accuracy of neural-network-based computational protein sequence design with DenseNet. J Chem Inf Model 2020;60:1245–52. 32126171 10.1021/acs.jcim.0c00043

[12] Zhang Y , Chen Y, Wang C, et al. Prodconn-protein design using a convolutional neural network. Biophys J 2020;118:43a–4. 10.1002/prot.25868 PMC8204568 31867753

[13] Strokach A , Becerra D, Corbi-Verge C, et al. Fast and flexible protein design using deep graph neural networks. Cell Syst 2020;11:402–411.e4. 32971019 10.1016/j.cels.2020.08.016

[14] Jing B , Eismann S, Suriana P, et al. Learning from protein structure with geometric vector perceptrons arXiv preprint arXiv:2009.01411. 2020.

[15] Anand N , Eguchi R, Mathews II, et al. Protein sequence design with a learned potential. Nat Commun 2022;13:746. 35136054 10.1038/s41467-022-28313-9 PMC8826426

[16] Liu Y , Zhang L, Wang W, et al. Rotamer-free protein sequence design based on deep learning and self-consistency. Nat Comput Sci 2022;2:451–62. 38177863 10.1038/s43588-022-00273-6

[17] Hsu C , Verkuil R, Liu J, et al. Learning inverse folding from millions of predicted structures. In: International Conference on Machine Learning. PMLR, 2022, 8946–70.

[18] Dauparas J , Anishchenko I, Bennett N, et al. Robust deep learning–based protein sequence design using ProteinMPNN. Science 2022;378:49–56. 36108050 10.1126/science.add2187 PMC9997061

[19] Zheng Z , Deng Y, Xue D, et al. Structure-informed language models are protein designers. bioRxiv 2023–02. 2023.

[20] Gao Z , Tan C, Li SZ. PiFold: toward effective and efficient protein inverse folding. arXiv preprint arXiv:2209.12643. 2022.

[21] Huang B , Fan T, Wang K, et al. Accurate and efficient protein sequence design through learning concise local environment of residues. Bioinformatics 2023;39:btad122. 36916746 10.1093/bioinformatics/btad122 PMC10027430

[22] Ying C , Cai T, Luo S, et al. Do transformers really perform badly for graph representation? Adv Neural Inf Process Syst 2021;34:28877–88.

[23] Rocklin GJ , Chidyausiku TM, Goreshnik I, et al. Global analysis of protein folding using massively parallel design, synthesis, and testing. Science 2017;357:168–75. 28706065 10.1126/science.aan0693 PMC5568797

[24] Verkuil R , Kabeli O, Du Y, et al. Language models generalize beyond natural proteins. bioRxiv 2022–12. 2022. 10.1101/2022.12.21.521521.

[25] Bahar I , Jernigan R, Dill K. In: Dill K, Jernigan RJ, Bahar I (eds.) Protein Actions: Principles and Modeling. New York: Garland Science, 2017.

[26] de los Santos YL , Chew-Fajardo YL, Brault G, Doucet N. Dissecting the evolvability landscape of the CalB active site toward aromatic substrates. Sci Rep 2019;9:15588. 31666622 10.1038/s41598-019-51940-0 PMC6821916

[27] Uppenberg J , Hansen MT, Patkar S, Jones TA. The sequence, crystal structure determination and refinement of two crystal forms of lipase B from Candida antarctica. Structure 1994;2:293–308. 8087556 10.1016/s0969-2126(00)00031-9

[28] Liu H , Chen Q. Computational protein design with data-driven approaches: recent developments and perspectives. Wiley Interdisciplinary Reviews. Comput Mol Sci 2023;13:e1646.

[29] Kao HW , Lu WL, Ho MR, et al. Robust Design of Effective Allosteric Activators for Rsp5 E3 ligase using the machine learning tool ProteinMPNN. ACS Synth Biol 2023;12:2310–9. 37556858 10.1021/acssynbio.3c00042

[30] Buel GR , Walters KJ. Can AlphaFold2 predict the impact of missense mutations on structure? Nat Struct Mol Biol 2022;29:1–2. 35046575 10.1038/s41594-021-00714-2 PMC11218004

[31] Wang J , Lisanza S, Juergens D, et al. Scaffolding protein functional sites using deep learning. Science 2022;377:387–94. 35862514 10.1126/science.abn2100 PMC9621694

[32] Li Z , Yang Y, Zhan J, et al. Energy functions in de novo protein design: current challenges and future prospects. Annu Rev Biophys 2013;42:315–35. 23451890 10.1146/annurev-biophys-083012-130315 PMC3851009

[33] Lobanov MY , Bogatyreva N, Galzitskaya O. Radius of gyration as an indicator of protein structure compactness. Mol Biol 2008;42:623–8. 18856071

[34] McGibbon RT , Beauchamp KA, Harrigan MP, et al. MDTraj: a modern open library for the analysis of molecular dynamics trajectories. Biophys J 2015;109:1528–32. 26488642 10.1016/j.bpj.2015.08.015 PMC4623899

[35] Zhang Y , Liu H, Yang S, et al. Well-balanced force field ff 03 CMAP for folded and disordered proteins. J Chem Theory Comput 2019;15:6769–80. 31657215 10.1021/acs.jctc.9b00623 PMC7196277

[36] Horn HW , Swope WC, Pitera JW, et al. Development of an improved four-site water model for biomolecular simulations: TIP4P-ew. J Chem Phys 2004;120:9665–78. 15267980 10.1063/1.1683075

[37] Mu J , Pan Z, Chen H-F. Balanced solvent model for intrinsically disordered and ordered proteins. J Chem Inf Model 2021;61:5141–51. 34546059 10.1021/acs.jcim.1c00407

[38] Pan Z , Mu J, Chen H-F. Balanced three-point water model OPC3-B for intrinsically disordered and ordered proteins. J Chem Theory Comput 2023;19:4837–50. 37452752 10.1021/acs.jctc.3c00297

[39] Wang J , Wang W, Kollman PA, Case DA. Automatic atom type and bond type perception in molecular mechanical calculations. J Mol Graph Model 2006;25:247–60. 16458552 10.1016/j.jmgm.2005.12.005

[40] Ester M , Kriegel H-P, Sander J, et al. A density-based algorithm for discovering clusters in large spatial databases with noise. in Kdd 1996;96:226–31.

[41] Jakalian A , Bush BL, Jack DB, Bayly CI. Fast, efficient generation of high-quality atomic charges. AM1-BCC model: I. Method. J Comput Chem 2000;21:132–46. 10.1002/jcc.10128 12395429

