# Supplementary material for: Graphormer supervised de novo protein design method and function validation
Source: Brief Bioinform. 2024 Mar 31;25(3):bbae135. doi: 10.1093/bib/bbae135 (PMC10982952; doi:10.1093/bib/bbae135)
Supplement: GPD_20240131_SI_re_bbae135 [file gpd_20240131_si_re_bbae135.docx]

# Supplementary Information

Graphormer Supervised *De Novo* Protein Design Method and Function Validation

Junxi Mu^1,2,†^, Zhengxin Li^1,†^, Bo Zhang^1,†^, Qi Zhang^1,†^, Jamshed Iqbal^3^, Abdul Wadood^4^, Ting Wei^1,*^, Yan Feng^1,*^, Hai-Feng Chen^1,*^

^1^State Key Laboratory of Microbial metabolism, Joint International Research Laboratory of Metabolic Developmental Sciences, Department of Bioinformatics and Biostatistics, National Experimental Teaching Center for Life Sciences and Biotechnology, School of Life Sciences and Biotechnology, Shanghai Jiao Tong University, 800 Dongchuan Road,
Shanghai, 200240, China.
^2^Center for Life Sciences, Academy for Advanced Interdisciplinary Studies, Peking University, No.5 Yiheyuan Road, Beijing, 100871, China.
^3^Centre for Advanced Drug Research, COMSATS University Islamabad, Abbottabad Campus, Abbottabad, 22060, Pakistan.
^4^Department of Biochemistry, Abdul Wali Khan University Mardan, Mardan, 23200, Pakistan.

† These authors contributed equally to this work

* Corresponding author(s). E-mail(s): haifengchen@sjtu.edu.cn; weitinging@sjtu.edu.cn; yfeng2009@sjtu.edu.cn;


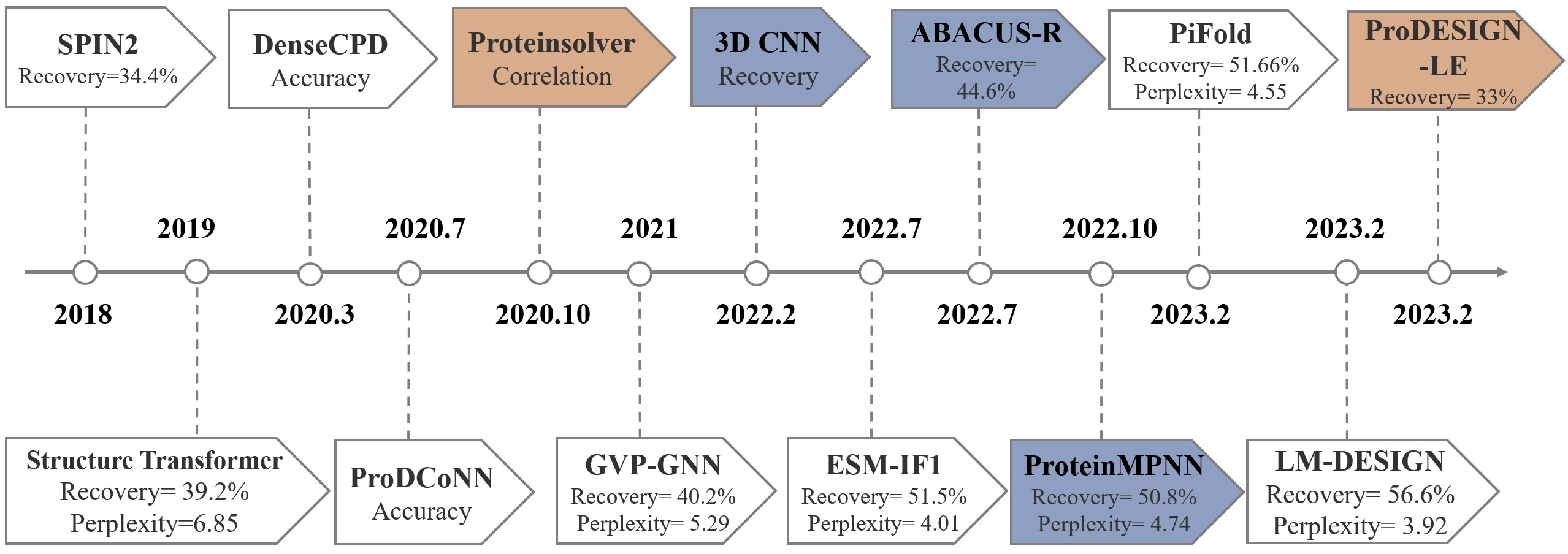


**Figure S1. The deep learning-based protein sequence design methods.**


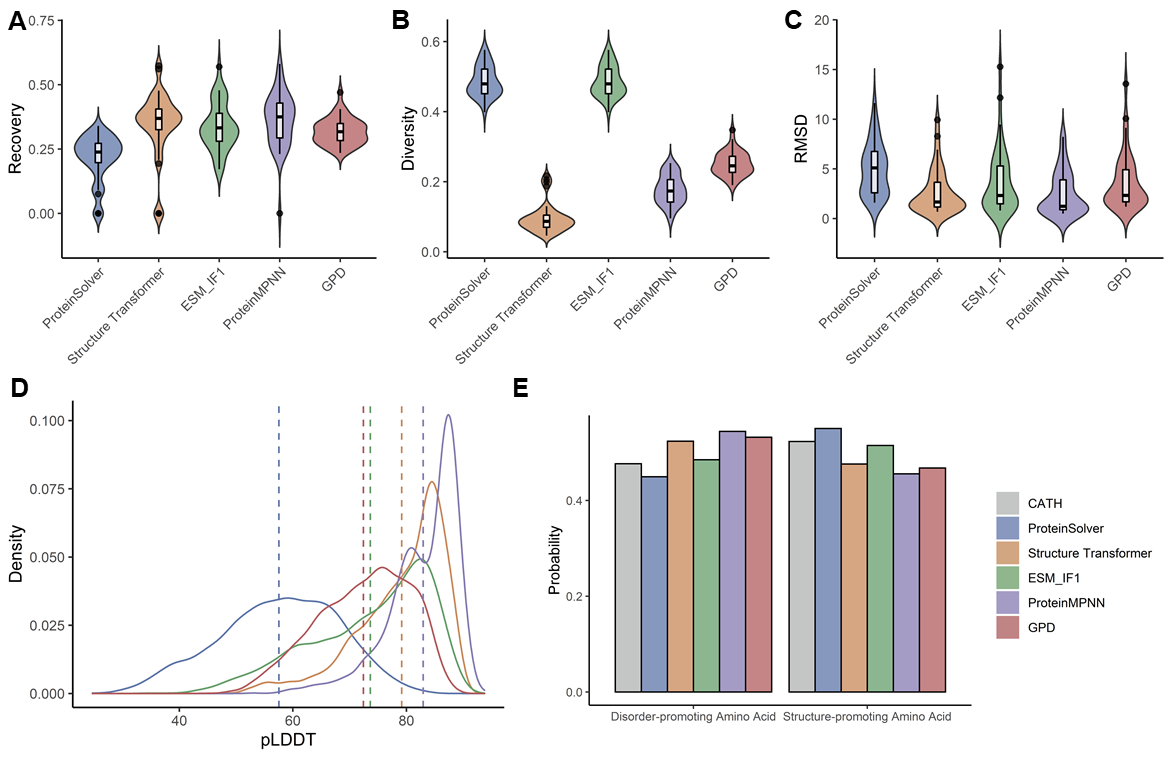


**Figure S2. Comparison of designed sequence for five methods on 39 de novo proteins. A:** the sequence recovery between the designed sequence and the native sequence of the target structure. **B:** The diversity of designed sequences. **C:** RMSD for aligning the ESMFold predicted structures with the corresponding native structures. **D:** The pLDDT scores of the ESMFold predicted structures. **E:** the frequency of disorder-promoting amino acids (alanine, glycine, proline, arginine, glutamine, serine, glutamic acid, and lysine) and structure-promoting amino acids (other twelve residues).


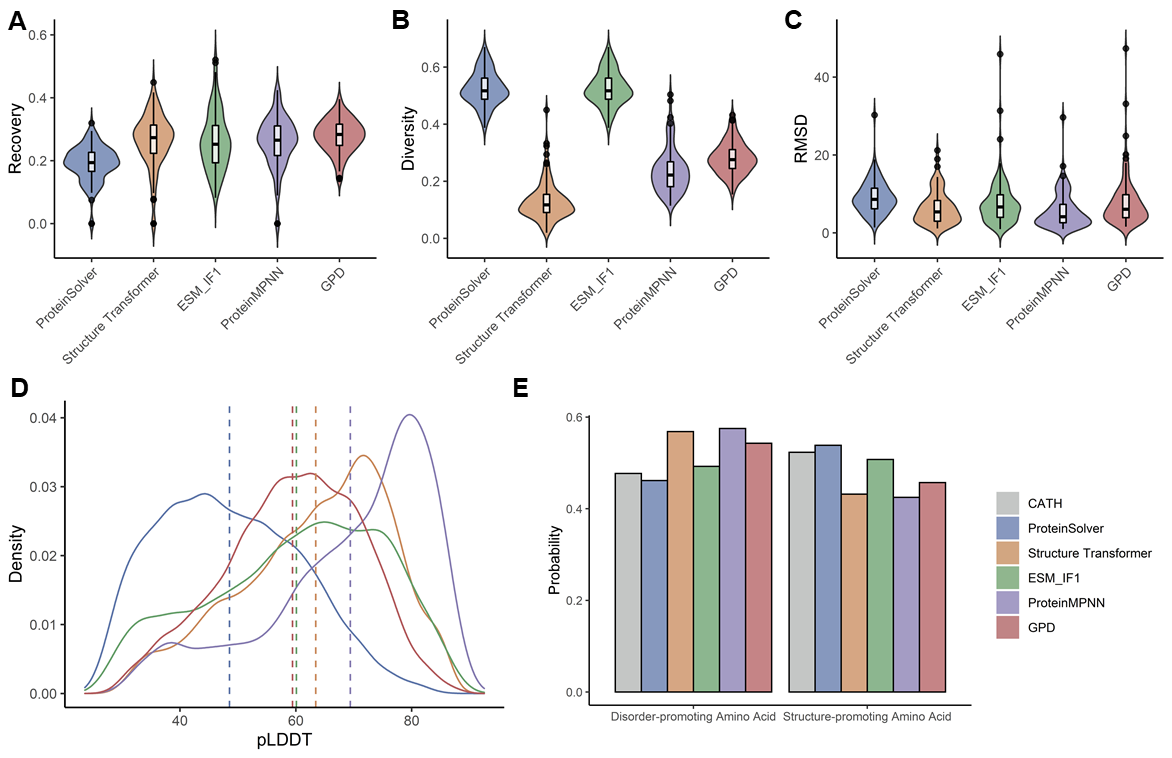


**Figure S3. Comparison of designed sequence for five methods on 103 single chain proteins. A:** the sequence recovery between the designed sequence and the native sequence of the target structure. **B:** The diversity of designed sequences. **C:** RMSD for aligning the ESMFold predicted structures with the corresponding native structures. **D:** The pLDDT scores of the ESMFold predicted structures. **E:** the frequency of disorder-promoting amino acids (alanine, glycine, proline, arginine, glutamine, serine, glutamic acid, and lysine) and structure-promoting amino acids (other twelve residues).


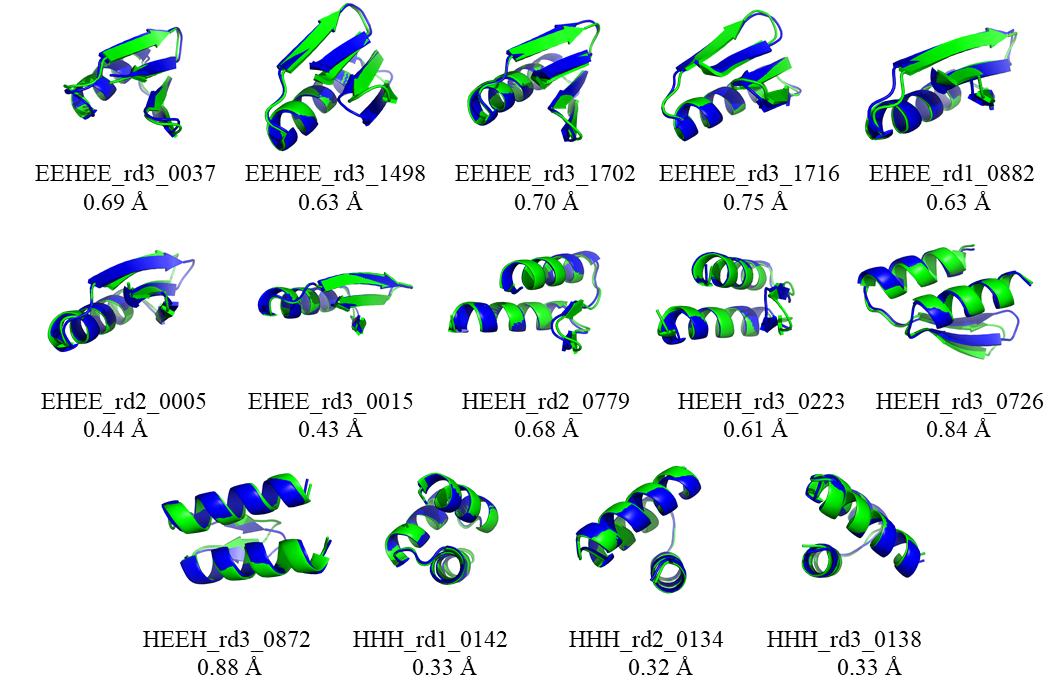


**Figure S4. Structure prediction by AlphaFold2 on designed sequences.** Overlay of native structures (green) with AlphaFold2 predicted structures of GPD model designed sequences (blue). The predicted structure with the minimum RMSD was shown.


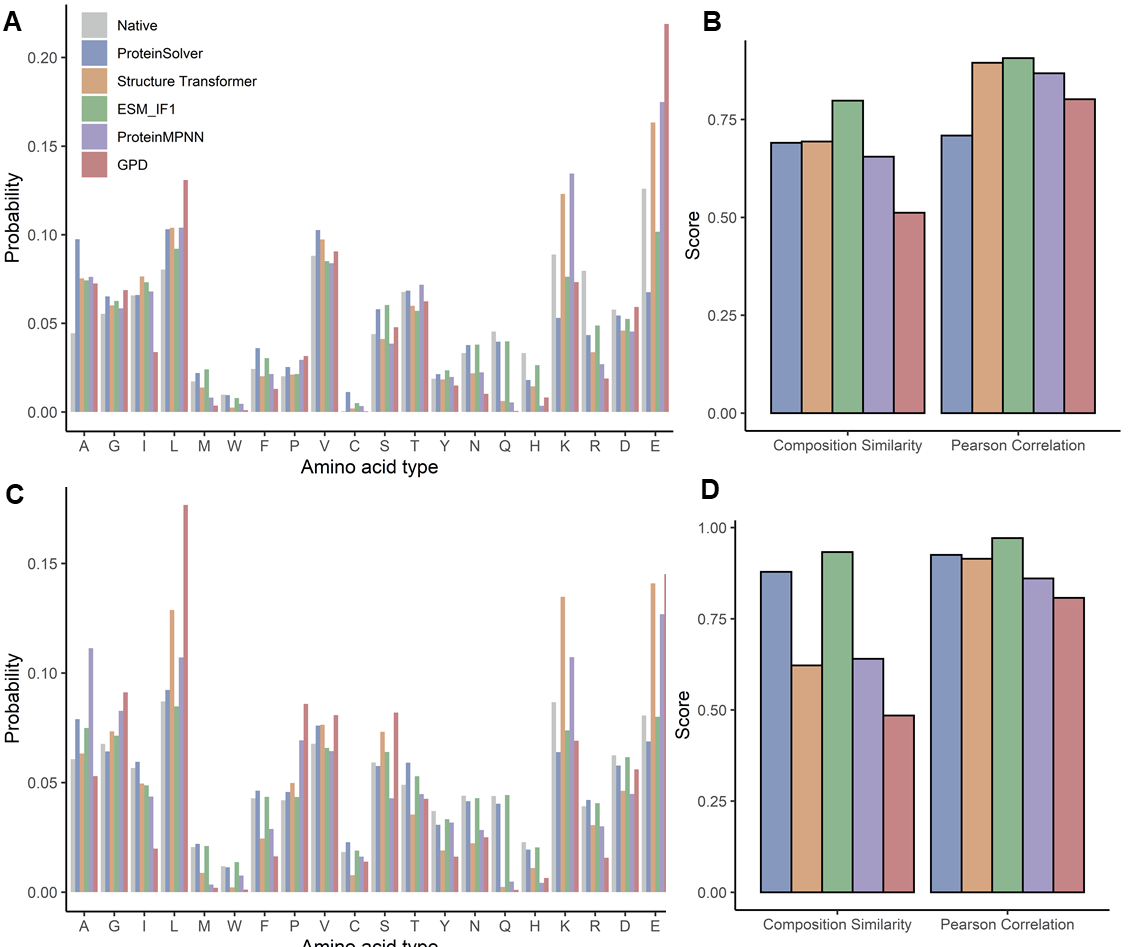


**Figure S5. The amino acids frequency of designed sequence on 39 de novo (A, B) and on 103 single chain proteins (C, D). A, C:** The sequence identity between the designed sequence and the native sequence of the target structure. **B, D:** The Pearson correlation coefficient and the composition similarity of the amino acid type compositions of the designed and the native sequences.


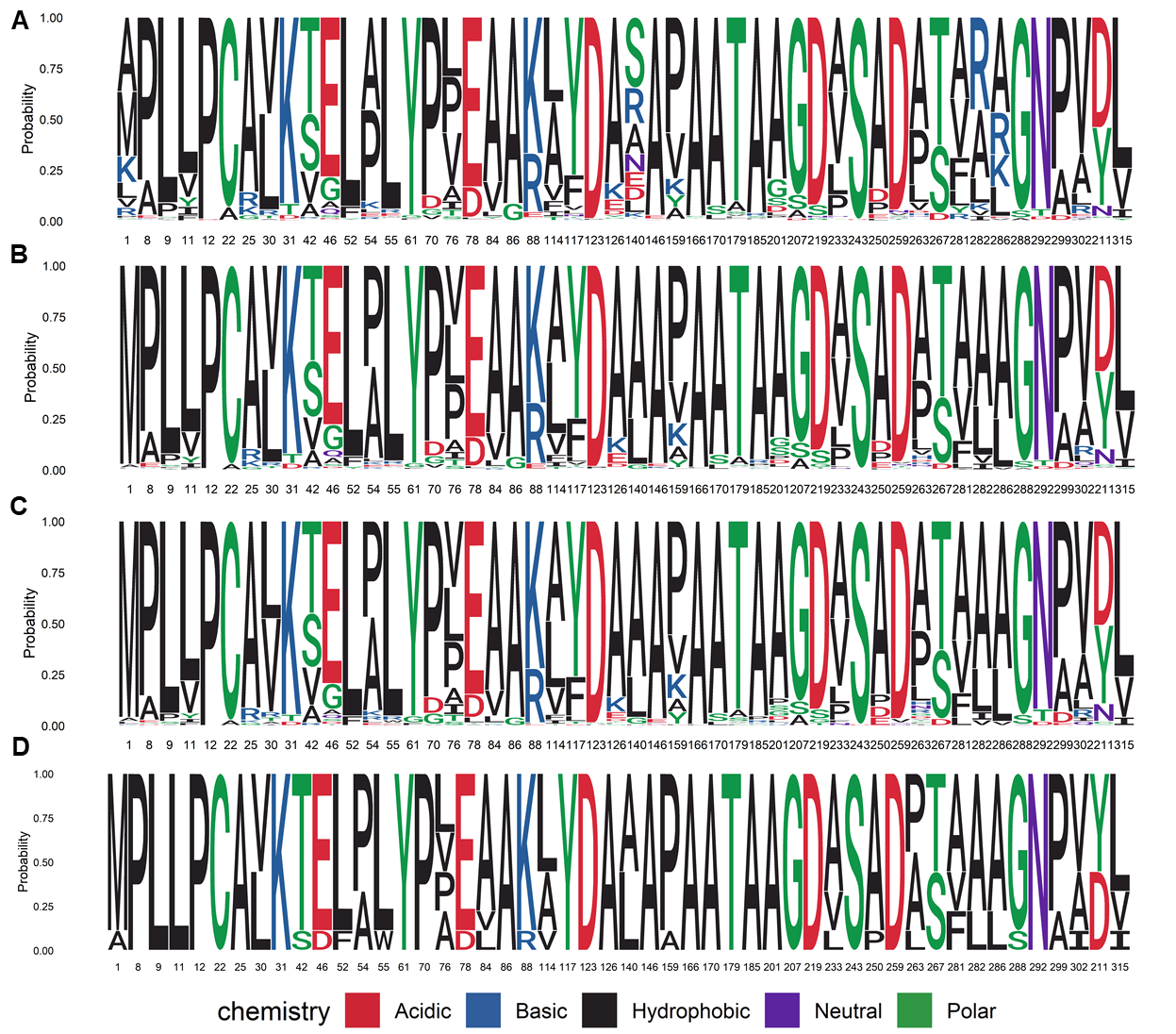


**Figure S6. The seqlog plot after each step screening. A:** The seqplot of 50 residues of 1,000,000 designed sequences of CalB. **B:** 485 sequences meet the meet the screening criteria of protein folding ability and protein solubility based on ESMFold. **C:** meet the meet the screening criteria of protein folding ability and protein solubility based AlphaFold2. **D:** The seqlog plot of 9 sequences meet the catalytic mechanism and were selected for experimental validation.


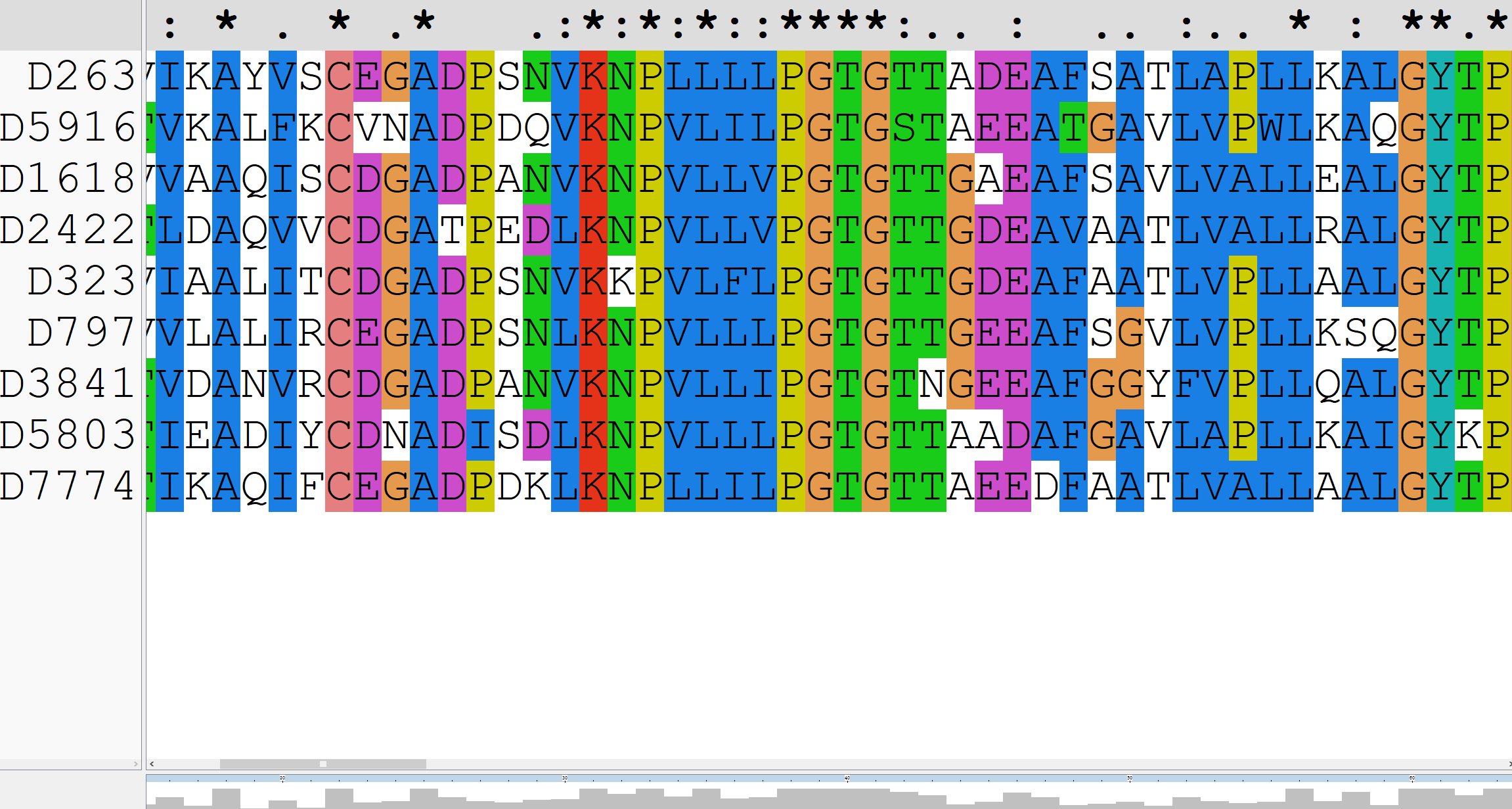


**Figure S7. The 9 sequences for experimental validation.**


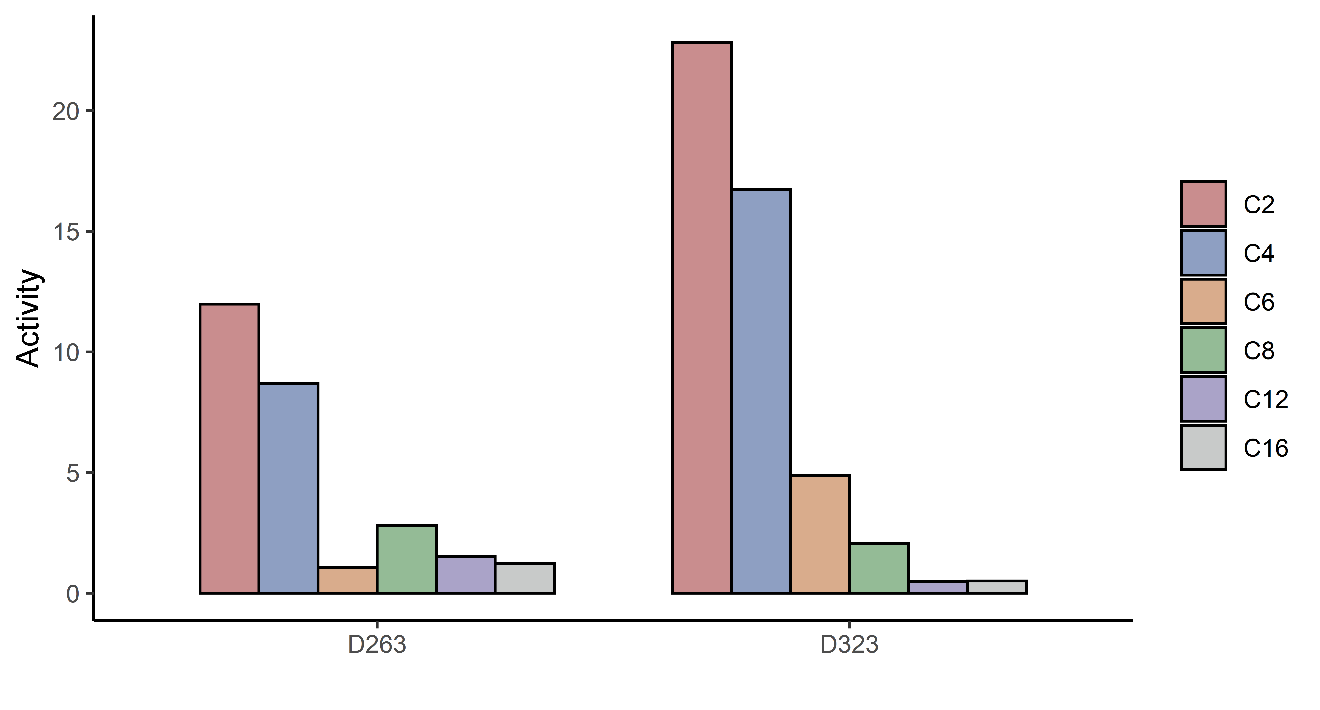


**Figure S8. The substrate selectivity of the designed sequences.**


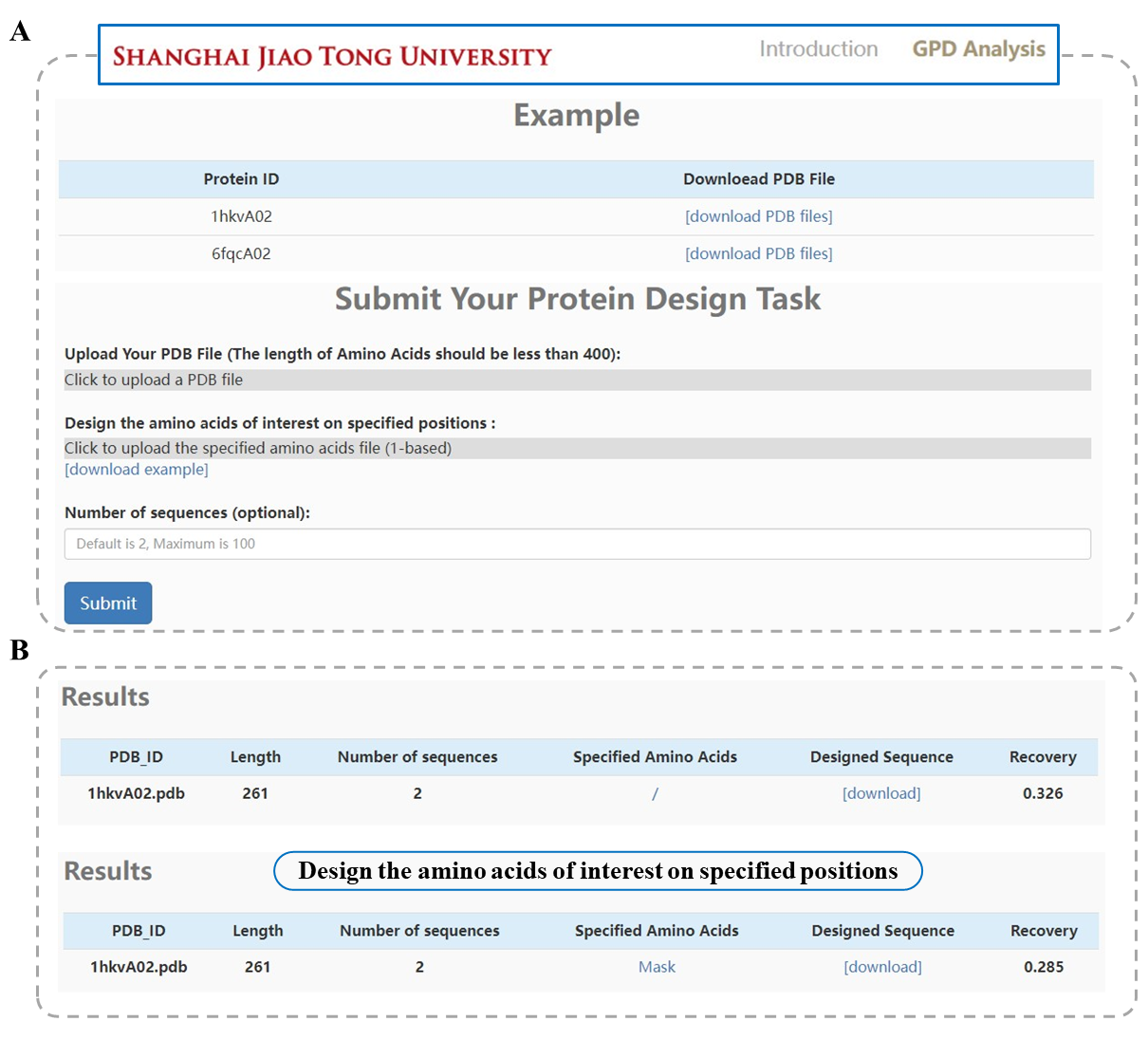


**Figure S9. The online tool for GPDGenerator. A:** The “Analysis” interface of the GPD web server. The user needs to input the PDB file and the specified amino acids (design the amino acids of interest at specified positions are optional. **B:** the example output of the web server.

**Table S1. The functional screening.**

| **The type of functional screening** | **Screening condition** | **# of sequences** |
| --- | --- | --- |
| CalB designed sequences | CalB designed sequences | 100w |
|  | Non-polar for 7 amino acides | 40278 |
| Protein folding ability (ESMFold) | RMSD < 1.5Å pLDDT > 80 | 824 |
|  | $Rg_{C}\alpha$ $\leq18.45Å$ | 793 |
| Protein solubility (ESMFold) | SAP $\leq182.421$ | 506 |
|  | Net Charge $\leq-1$ | 506 |
|  | Surface Polar Score $\geq0.450$ | 485 |
| Protein folding ability (AlphaFold2) | RMSD < 1.5Å pLDDT > 80 | 467 |
|  | Self-consistent < 2Å | 184 |
|  | $Rg_{C}\alpha$ $\leq18.45Å$ | 180 |
| Protein solubility (AlphaFold2) | SAP $\leq182.421$ | 152 |
|  | Net Charge $\leq-1$ | 152 |
|  | Surface Polar Score $\geq0.450$ | 151 |
| MD simulations | Catalytic mechanism | 9 |
|  |  |  |
